# Atrial Fibrillation on Patients with Vascular Dementia: A Fundamental Target for Correct Management

**DOI:** 10.3390/brainsci10070420

**Published:** 2020-07-02

**Authors:** Giovanna Viticchi, Lorenzo Falsetti, Marco Burattini, Vincenzo Zaccone, Laura Buratti, Marco Bartolini, Gianluca Moroncini, Mauro Silvestrini

**Affiliations:** 1 Neurological Clinic, Marche Polytechnic University, 60020 Ancona, Italy; marco.burattini1@gmail.com (M.B.); lauraburatti2016@gmail.com (L.B.); m.bartolini@univpm.it (M.B.); m.silvestrini@univpm.it (M.S.); 2 Internal and Subintensive Medicine, Ospedali Riuniti Ancona, 60020 Ancona, Italy; drfalsetti@yahoo.it (L.F.); vincenzozaccone@libero.it (V.Z.); 3 Department of Clinical and Molecular Sciences, Marche Polytechnic University, 60020 Ancona, Italy; g.moroncini@univpm.it

**Keywords:** vascular dementia, atrial fibrillation, anticoagulants, CHA_2_DS_2_-VAS score, HAS-BLED score

## Abstract

Background: Atrial fibrillation (AF) is a risk factor for cerebrovascular diseases and vascular dementia (VAD). The aim of this study was to evaluate the effect of the adherence to anticoagulant therapy guidelines in patients with dementia and AF on the risk of stroke/TIA or major bleeding (MB). Methods: In a cohort of 1705 hospitalized patients with pre-existent AF, we observed 193 patients with vascular dementia (VAD). Non-demented AF patients were included as controls. For each subject, we calculated CHA_2_DS_2_-VASc, CHADS_2_, and HAS-BLED scores, and collected information regarding anticoagulant therapy, in-hospital therapeutic failure (TF) occurrence, stroke/TIA, and MB. Results: According to CHA_2_DS_2_-VASc and CHADS_2_ scores, 99.5% of VAD patients had the indication to anticoagulant treatment, but only 69.9% were correctly treated. During hospitalization, MB occurred in 4.66% of VAD and 8.9% of non-demented patients (*p* = 0.048). In-hospital stroke/TIA were observed in 24.3% of VAD and 0.8% of non-demented patients (*p* = 0.0001). A similar proportion of TF among patients with VAD and with normal cognition (12.9% vs. 11.2%) was observed. Conclusion: In our cohort, we observed that VAD patients with pre-existent AF were undertreated despite a higher risk of stroke/TIA with respect to non-demented patients.

## 1. Introduction

Vascular dementia (VAD) is usually associated with several vascular risk factors such as hypertension, insulin resistance, diabetes, obesity, hyperhomocysteinemia, and dyslipidemia [1,2,3]. Recent evidence shows a narrow relationship between atrial fibrillation (AF) and different types of dementia [4,5,6]. Particularly, AF seems to be strictly associated with both Alzheimer’s disease (AD) and vascular dementia [7]. The Rotterdam study showed that AF is correlated with all causes of dementia, especially when present for several years [8].

AF is one of the most relevant causes of severe stroke. Several studies showed that cognitive decline in AF patients is mainly associated with silent cerebral infarctions due to a probably undetected embolic mechanism [4,9]. Another possible pathogenic hypothesis concerns a reduced cerebral blood flow for a lowered cardiac output with a consequent chronic cerebral hypoperfusion [4,10]. Regarding the impact of therapy, there is evidence underlining that a correct anticoagulation in AF patients seems to decrease the risk of dementia [11,12]. Further, anticoagulants are more effective than antiplatelet drugs in dementia prevention [13].

In spite of this evidence, very often elderly patients with AF, who are at the highest risk for vascular diseases and cognitive impairment, are not correctly treated with anticoagulants [14].

The aim of this study was to evaluate, in AF patients affected by VAD, the adherence to the therapeutic indication for anticoagulation according to the current guidelines, its correlation with ischemic and hemorrhagic risk, and the outcome after hospitalization. We also evaluated non-demented patients with AF to compare the characteristics of these two different populations.

## 2. Materials and Methods

### 2.1. Study Cohort

For this study, we employed data from the AFICILL (Atrial Fibrillation In Critically ILl) study. AFICILL participants belonged to a retrospective cohort of consecutive patients analyzed in the timeframe January 2002–March 2008. In our study, were enrolled patients with pre-existing AF. Patients were risk-stratified and treated in accordance with the ACC/AHA/ESC 2006 guidelines, which were the most updated at the time of the study [15]. However, we also re-stratified and re-evaluated the risk of our subjects in accordance with the 2016 ESC guidelines in order to give more complete information [16]. Our patients were affected by a critical illness requiring an urgent admission from the emergency department to the stepdown unit (SDU) of the internal and sub-intensive medicine department of the University Hospital “Ospedali Riuniti” of Ancona, Italy. The study was authorized by the institutional review board (Prot. 168/2018, June 2018). All patients were treated in accordance with the current guidelines and accordingly with the declaration of Helsinki.

For each patient we collected age, sex, and comorbidities, evaluating the presence of chronic heart failure (CHF), hypertension (HTN), peripheral artery disease (PAD), previous stroke/TIA, previous gastrointestinal bleeding, type 2 diabetes mellitus (T2DM), chronic obstructive pulmonary disease (COPD), coronary artery disease (CAD), chronic kidney disease (CKD), chronic hepatic diseases (CHD), chronic anemia, and active cancer. At the admission, the thromboembolic risk was estimated with both CHA_2_DS_2_-VASc and CHADS_2_, while the hemorrhagic risk was calculated with HAS-BLED, following the original definition of each score [17]. Patients were considered “in anticoagulant therapy” only if they were treated with warfarin or low-molecular weight heparin before the hospital admission. Stroke and major bleeding were evaluated as complicating conditions of a critical illness leading to the admission of the patients.

According to medical history and previously reported neuropsychological examinations, we collected patients affected by different forms of dementia. For this study, we chose to analyze only subjects with a diagnosis of VAD according to American Heart Association/American Stroke Association Diagnostic criteria [18].

We also evaluated in-hospital death or intensive care unit (ICU) transfer, which were synthesized as a binary variable named therapeutic failure (TF). In-hospital occurrence of stroke/TIA and major bleeding (MB) following the hospital admission were also retrospectively collected. We did not differentiate among various typologies of stroke and TIA because we considered that this datum did not impact significantly on the study design objectives and would have introduced an excessive number of patient subgroups.

### 2.2. Statistical Analysis

The presence of any form of dementia, presence of VAD, sex, TF, in-hospital occurrence of stroke/TIA, in-hospital occurrence of MB, and anticoagulant and antiplatelet drugs use at the admission and all the collected comorbidities were synthesized as binary variables. Age, CHA_2_DS_2_-VASc, CHADS_2_, and HAS-BLED scores were collected as continuous variables.

CHA_2_DS_2_-VASc was transformed into a dichotomous variable in order to classify patients at low thromboembolic risk (CHA_2_DS_2_-VASc = 0–1) or high thromboembolic risk (CHA_2_DS_2_-VASc ≥ 2), in accordance with current guidelines [16]. Similarly, CHADS_2_ was recoded in a binary variable to classify patients at low thromboembolic risk (CHADS_2_ = 0–1) or high thromboembolic risk (CHADS_2_ ≥ 2), in accordance with current guidelines [16]. HAS-BLED score was synthesized into a binary variable to discriminate between patients at low hemorrhagic risk (HAS-BLED = 0–2) and high hemorrhagic risk (HAS-BLED ≥ 3), in accordance with current guidelines [16].

Dichotomous and categorical variables were presented as the absolute number and percent and compared with a chi-squared test. Continuous variables were tested for normality with the Kolomogorov–Smirnov test. Normally distributed variables were presented as mean and standard deviation (SD) and compared with a *t*-test for independent measures or ANOVA. Non-normally distributed variables were presented as the median and interquartile range (IQR) and compared with the Mann–Whitney U test or Kruskal–Wallis test. The correlation between variables was evaluated with Pearson’s bivariate test. We performed three logistic regression models adopting, respectively, TF, in-hospital stroke/TIA, and in-hospital MB as dependent variables. The covariates were selected from Pearson’s bivariate test if associated with the dependent variable at a level of *p* ≤ 0.10. We considered significant all the other comparisons and results at a level of *p* ≤ 0.05. The statistical analysis was performed with SPSS 13.0 for Windows systems.

## 3. Results

We obtained a final sample of 1705 consecutive patients admitted to the SDU of our hospital and affected by pre-existing AF. Of the patients, 1468 (86%) were not affected by any form of dementia (No-DEM), while 193 (11.31%) were affected by VAD. A heterogeneous group of 44 (2.6%) subjects, affected by other types of dementia (AD, Parkinson-dementia, and other forms of cognitive impairment), were not analyzed separately. Baseline differences between No-DEM and VAD subjects are synthesized in Table 1.

Patients with any form of dementia (DEM) had a significantly higher age (84.32 ± 6.81) than No-DEM subjects (76.65 ± 11.12, *p* = 0.0001 at *t*-test). Males represented 38.8%, while females were 61.2% of DEM subjects (*p* = 0.0001 at chi-squared test). 

DEM patients showed a significantly higher proportion of anticoagulant use (67.9%) than that of No-DEM subjects (60.1%, *p* = 0.021 at chi-squared test). Patients affected by VAD were significantly more treated (69.9%) than No-DEM subjects (60.1%, *p* = 0.008 at chi-squared test). Antiplatelet agent use, alone or in combination with anticoagulants, was similar in DEM (38.0%) and in No-DEM (37.5%, *p* = 0.881 at chi-squared test) subjects, with no significant difference in the VAD group (*p* = 0.427 at chi-squared test).

Adopting the CHA_2_DS_2_-VASc score to stratify the thromboembolic risk, we observed that 99.5% of DEM subjects were at high risk according to current guidelines (score ≥ 2), and that this proportion was significantly higher when compared with No-DEM patients (88.0%, *p* = 0.0001 at chi-squared test). Considering the CHADS_2_ score, we observed that 90.3% of DEM patients were at high risk (score ≥ 2). This percentage was significantly higher when compared to that of No-DEM patients (58.0%, *p* = 0.0001 at chi-squared test). A high bleeding risk, defined as a HAS-BLED score ≥ 3, was significantly more prevalent among DEM subjects (60.8%) with respect to No-DEM patients (35.9%, *p* = 0.0001 at chi-squared test). When analyzing the subgroups, we observed that the largest part of subjects with VAD (99.5%) had a CHA_2_DS_2_-VASc ≥ 2. In non-demented patients the percentage was significantly lower (88.0%, *p* = 0.0001 at chi-squared test). CHADS_2_ was ≥ 2 in 92.7% of patients with VAD, while in No-DEM subjects the proportion of cases at high-risk according to this score was significantly lower (58.0%, *p* = 0.0001 at chi-squared test).

**Figure 1:**
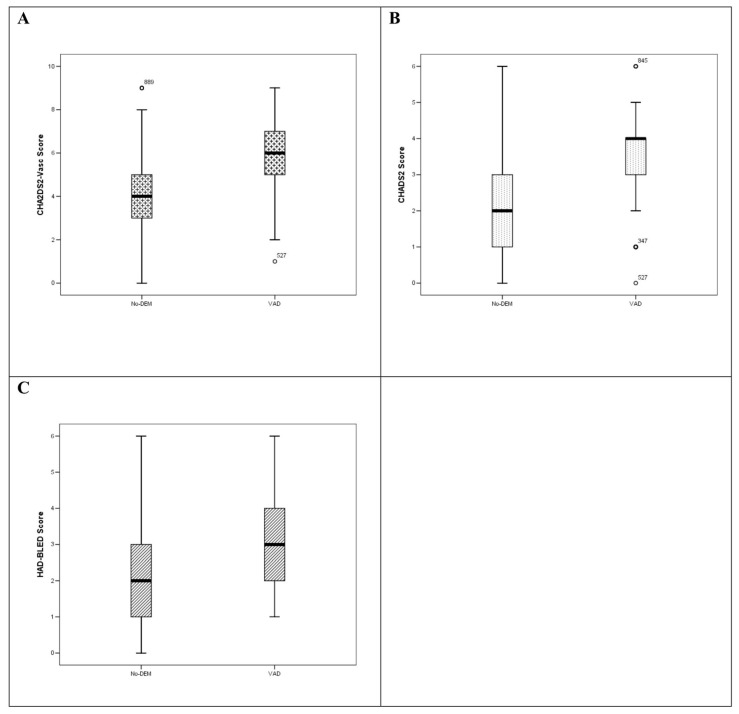
Differences in vascular risk scores between No-DEM and VAD. (**A**) CHA_2_DS_2_-VASc score was significantly lower in No-DEM than in VAD patients; (**B**) CHADS_2_ score was significantly lower in No-DEM than in VAD patients; (**C**) HAS-BLED score was significantly higher in patients with VAD than in No-DEM subjects.

Of note, CHA_2_DS_2_-VASc and CHADS_2_ scores were significantly lower in No-DEM patients than in VAD (Table 2, Figure 1A,B). The HAS-BLED score was significantly higher in patients with VAD than in No-DEM subjects (Table 2, Figure 1C).

During hospitalization, we did not observe a significantly higher prevalence of major bleeding (MB) in DEM patients (6.8%) when compared with No-DEM subjects (8.9%, *p* = 0.283 at chi-squared test). On the other hand, MB was significantly more frequent in No-DEM than in VAD patients (4.66% vs. 8.9, *p* = 0.048 at chi-squared test). 

DEM patients had a significantly higher prevalence of in-hospital occurrence of stroke/TIA after SDU admission (22.4%) when compared to No-DEM patients (10.8%, *p* = 0.00001 at chi-squared test). Among DEM subjects, VAD had the highest proportions of cases (24.3%) and this difference was statistically significant (*p* = 0.0001 at chi-squared test).

In this cohort, patients admitted to SDU with any form of dementia were not at higher risk of TF (14.8%) when compared with patients with normal cognition (11.2%, *p* = 0.110 at chi-squared test). Similarly, we observed a similar TF proportion among patients with VAD (12.9%) and normal cognition (11.2%, *p* = 0.464 at chi-squared test).

Pearson’s bivariate test confirmed the association between DEM and the in-hospital occurrence of stroke/TIA (*p* = 0.0001), though it did not confirm a significant relationship between DEM and TF (*p* = 0.283) or the in-hospital occurrence of MB (*p* = 0.110). 

In accordance with the data analysis plan, we performed a logistic regression analysis considering in-hospital stroke/TIA occurrence as a dependent variable, and DEM, binary CHA2DS2-VASc, anticoagulant therapy at the admission, and COPD as independent variables. The other variables associated with this outcome at Pearson’s bivariate test (age, sex, CHF, and previous stroke/TIA) were not included in the model because they were already considered in the CHA_2_DS_2_-VASc score. According to the first model’s results, we observed that (i) the presence of any form of dementia was associated with an increased risk of stroke/TIA during the following hospitalization (OR: 2.071; 95%CI: 1.455–2.948; *p* < 0.001), (ii) the absence of anticoagulant therapy at the admission increased the stroke/TIA risk (OR: 1.754;95%CI: 1.270–2.422; *p* < 0.001), (iii) a high thromboembolic risk (CHA2DS2-VASc ≥ 2) was associated with a higher risk of in-hospital stroke/TIA (OR: 2.734; 95%CI: 1.365–5.477), and (iv) patients with COPD had a lower risk of stroke/TIA following hospitalization (OR: 0.681; 95%CI: 0.470–0.988; *p* = 0.043). When considering specifically VAD patients, we observed that this type of dementia (*p* = 0.001) was significantly associated with an increased stroke/TIA risk during the following SDU hospitalization (Table 3).

## 4. Discussion

Our study underlined several critical points regarding the management of patients with VAD and pre-existent AF. AF is present in a large proportion of patients but very often, although there is a high risk for acute or chronic cerebrovascular diseases, a significant rate of cases are not correctly treated [19].

In our study, we observed that, among VAD patients, only 69.9% were correctly treated with anticoagulants before hospital admission. According to current guidelines, based on CHA_2_DS_2_-VASc or CHADS_2_ scores, more than 90% of these patients had an indication for anticoagulant treatment. In our study sample, No-DEM patients were also undertreated, but the rate of inappropriate therapy was less remarkable than in VAD patients. Further, VAD patients were at a higher risk of acute cerebrovascular diseases during hospitalization, since they underwent a new stroke/TIA in 24.3% of the cases, a percentage significantly higher than that observed in No-DEM patients.

CHA_2_DS_2_-VASc/CHADS_2_ scores were probably higher in VAD because previous stroke/vascular disease are very common in these patients. Moreover, other parameters which are common among older people, such as diabetes or hypertension, could have played a significant weight on these scores calculation, so we considered CHADS_2_ 2-VASc and CHADS_2_ as good markers of global and vascular assessment of our patients. Elderly patients with AF are at particular risk of in-hospital stroke [20], which could lead to major complications such as prolonged hospitalization, infections, possible heart failure, and reduced quality of life [14,21]. For this reason, they are expected to benefit most from anticoagulation [14]. Considering AF patients with dementia, several studies indicated that a correct anticoagulant therapy could be beneficial for both stroke risk and cognitive impairment progression [22].

There are several possible explanations for the efficacy of anticoagulation in reducing cognitive impairment progression. The most obvious effect of anticoagulation is the reduced risk of cerebral damage caused by thrombo and micro-embolism [11]. In this respect, cognitive impairment in AF patients has been related to silent infarcts due to micro-embolism or occult embolization. Inflammation is another AF-related mechanism correlated with cognitive deterioration. In this respect, inflammation is associated with different conditions such as oxidative stress, apoptosis, and cardiac fibrosis, which could promote different arrhythmias including AF. On the other hand, the inflammatory process induces endothelial dysfunction, platelet activation, and coagulation cascade activation [23]. Anticoagulants could indirectly reduce the effects of inflammation on the coagulation cascade. A higher level of hemostatic activation markers in AF patients has been demonstrated. Accordingly, the use of warfarin could reduce the risk of cognitive deterioration [24]. The reduction of the risk of dementia in AF subjects treated with anticoagulants could decrease up to 20% of the potential cases of AF-related cognitive impairment [11]. Even though the results of different studies about this issue were not univocal [25,26], the protective effect of anticoagulants seems to be evident [12,24,27]. With a correct treatment, it could then be possible to obtain a contemporary effective major protection both for stroke and cognitive impairment [28].

Some studies showed that the protective effect of anticoagulation is more pronounced in patients at high cardiovascular risk as measured by a CHA_2_DS_2_-VASc score ≥ 2) [11]. Among our patients, 99.5% of VAD subjects presented a CHA_2_DS_2_-VASc ≥ 2. 

Elderly patients, especially those affected by dementia, are often undertreated [19,29]. This is probably due to the fact that this population is usually considered frail and at high risk of bleeding. Furthermore, trials very often tend to exclude elderly or demented subjects and, for this reason, data on these groups are missing [19,30].

In our study, VAD patients presented a significantly higher HAS-BLED score than No-DEM subjects. Despite this, VAD patients had a lower rate of MB during the hospitalization and a higher stroke incidence. Moreover, they did not present a significant difference in therapeutic failure with respect to non-demented patients. These data confirm the need for a correct therapeutic approach in these patients. A consensus statement reported that anticoagulant drugs were associated with major protective effects in older subjects than younger subjects if they presented a higher bleeding risk [31]. Moreover, some studies showed that, especially in elderly subjects, anticoagulants did not imply a higher rate of bleeding and a worse tolerability with respect to high-dose aspirin [29,32].

Regarding cognitively impaired elderly patients, different findings suggested that the bleeding risk is similar for demented and non-demented subjects [19]. On the other hand, among VAD subjects the risk of ischemic cerebrovascular disease is 3 to 5 times higher than the bleeding risk [19,33]. Thus, a correct anticoagulation should be strongly suggested. Obviously, to minimize bleeding risk in this population, a narrow supervision is required. Low adherence is one of the most relevant problems in AF/demented patients [34,35]. AF subjects living alone or without regular assistance, especially if affected by dementia, were more often undertreated or had a low adherence [34].

This study has some limitations. First, this is a retrospective investigation of a real-life population. Further, the cohort was not originally designated for research purposes. Anticoagulation in our patients was obtained with low-molecular weight heparin or warfarin. Direct oral anticoagulants were not in use in Italy at the time of the study, so we could not have data available for direct comparison among the three typologies of anticoagulants. However, we considered that the therapies employed in the study are currently largely used, especially in older people with AF, because patients very often present contraindications for DOAC use. For all these reasons, since they have a different risk profile, further studies are required to assess the effects of DOACs in this specific population. We hope in the future to update our considerations with more recent data. Moreover, we considered the treatment at admission and the events occurring during the hospitalization, but we were not able to perform a long-term follow-up to evaluate the progression of the cognitive status. Further, we did not obtain data about the clinical and cognitive outcome after discharge and this was mainly due to the sample dimension.

## 5. Conclusions

According to our study results, VAD patients with pre-existent AF were undertreated despite a higher risk of stroke/TIA. Thus, a correct anticoagulation should be considered in the global management of patients with AF. This could be particularly of interest in patients also affected by VAD.

## Figures and Tables

**Table 1:** Baseline differences between patients not affected by any form of dementia (No-DEM) and vascular dementia (VAD) subjects.

	Overall (*n* = 1705)	No-DEM (*n* = 1468)	VAD (*n* = 193)	P
General characteristics				
Age (mean ± SD)	77.7 ± 10.9	76.6 ± 11.2	84.3 ± 6.85	0.000
Male sex (n, %)	862 (50.6%)	770 (52.4%)	77 (39.8%)	0.0001
Main outcomes				
TF (n, %)	199 (11.6%)	164 (11.2%)	25 (12.9%)	0.464
Stroke/TIA (n, %)	211 (12.4%)	158 (10.7%)	47 (24.3%)	0.0001
MB (n, %)	146 (8.6%)	130 (8.9%)	9 (4.7%)	0.048
Comorbidities				
Hypertension (n, %)	838 (47.8%)	728 (49.6%)	89 (4.6%)	0.364
T2DM (n, %)	284 (16.6%)	256 (17.4%)	22 (11.4%)	0.035
CAD (n, %)	657 (38.5%)	567 (38.6%)	78 (40.4%)	0.631
CHF (n, %)	693 (40.6%)	589 (40.1%)	82 (42.4%)	0.529
PAD (n, %)	153 (8.9%)	138 (9.4%)	13 (6.7%)	0.226
Previous stroke/TIA (n, %)	300 (17.1%)	110 (7.5%)	166 (86.0%)	0.0001
CHD (n, %)	46 (2.7%)	41 (2.8%)	4 (2.1%)	0.562
CKD (n, %)	283 (16.6%)	250 (17.0%)	24 (12.4%)	0.106
Chronic anemia (n, %)	135 (7.91%)	114 (7.8%)	14 (7.2%)	0.802
Active cancer (n, %)	279 (16.4%)	246 (16.7%)	28 (14.5%)	0.429
COPD (n, %)	412 (24.2%)	369 (25.1%)	35 (18.1%)	0.033
Risk scores				
CHA_2_DS_2_VASc (mean, ±SD)	3.93 ± 1.8	3.68 ± 1.7	5.69 ± 1.4	0.0001
CHADS_2_ (mean, ±SD)	2.11 ± 1.4	1.89 ± 1.3	3.65 ± 1.2	0.0001
HAS-BLED (mean, ±SD)	2.21 ± 1.1	2.11 ± 1.1	2.93 ± 1.0	0.0001
Treatment				
Anticoagulants (n, %)	1043 (61.2%)	882 (60.1%)	135 (69.9%)	0.008
Antiplatelet drugs (n, %)	640 (37.5%)	550 (37.4%)	78 (40.4%)	0.427

Legend: SD = standard deviation; TF = therapeutic failure; TIA = transient ischemic attack; MB = major bleeding; T2DM = type 2 diabetes mellitus; CAD = coronary artery disease; CHF = chronic heart failure; PAD = peripheral artery disease; CHD = chronic hepatic diseases; CKD = chronic kidney disease; COPD = chronic obstructive lung disease.

**Table 2:** Comparison between subgroups of patients affected by atrial fibrillation (AF).

Dependent Variable	Mean (I)	Mean (J)	Mean Difference (I−J)	SE	P
CHA_2_DS_2_-Vasc	No-DEM	VAD	−2.009	0.130	0.0001
CHADS_2_	No-DEM	VAD	−1.763	0.097	0.0001
HAS-BLED	No-DEM	VAD	−0.820	0.083	0.0001

Legend: SE = standard error; No-DEM = non-demented subjects; VAD = vascular dementia.

**Table 3:** Logistic regression analysis results considering in-hospital stroke/TIA as dependent variable.

	P	OR	95% CI	
			Lower	Upper
**No dementia (Ref.)**	0.001			
**VAD**	0.001	2.282	1.569	3.319
**CHA_2_DS_2_VASc ≥ 2**	0.005	2.711	1.353	5.432
**No anticoagulant at admission**	0.001	1.745	1.257	2.424
**COPD**	0.028	0.756	0.447	0.956
**Constant**	0.000			

Legend: COPD = chronic obstructive lung disease; VAD = vascular dementia.

## References

[1] Viticchi G., Falsetti L., Buratti L., Sajeva G., Luzzi S., Bartolini M., Provinciali L., Silvestrini M. (2017). Framingham Risk Score and the Risk of Progression from Mild Cognitive Impairment to Dementia. J. Alzheimer’s Dis..

[2] Silvestrini M., Viticchi G., Altamura C., Luzzi S., Balucani C., Vernieri F. (2012). Cerebrovascular Assessment for the Risk Prediction of Alzheimer’s Disease. J. Alzheimer’s Dis..

[3] Viticchi G., Falsetti L., Buratti L., Luzzi S., Bartolini M., Acciarri M.C., Provinciali L., Silvestrini M. (2015). Metabolic syndrome and cerebrovascular impairment in Alzheimer’s disease. Int. J. Geriatr. Psychiatry.

[4] Diener H.-C., Hart R.G., Koudstaal P.J., Lane D.A., Lip G.Y. (2019). Atrial Fibrillation and Cognitive Function: JACC Review Topic of the Week. J. Am. Coll. Cardiol..

[5] Santos C.Y., Snyder P.J., Wu W.-C., Zhang M., Echeverria A., Alber J. (2017). Pathophysiologic relationship between Alzheimer’s disease, cerebrovascular disease, and cardiovascular risk: A review and synthesis. Alzheimer’s Dement. Diagn. Assess. Dis. Monit..

[6] Dietzel J., Haeusler K.G., Endres M. (2017). Does atrial fibrillation cause cognitive decline and dementia?. Europace.

[7] Falsetti L., Viticchi G., Buratti L., Grigioni F., Capucci A., Silvestrini M. (2018). Interactions between Atrial Fibrillation, Cardiovascular Risk Factors, and ApoE Genotype in Promoting Cognitive Decline in Patients with Alzheimer’s Disease: A Prospective Cohort Study. J. Alzheimer’s Dis..

[8] De Bruijn R.F.A.G., Heeringa J., Wolters F.J., Franco O.H., Stricker B.H., Hofman A., Koudstaal P.J., Ikram M.A. (2015). Association Between Atrial Fibrillation and Dementia in the General Population. JAMA Neurol..

[9] Chen L.Y., Lopez F.L., Gottesman R.F., Huxley R.R., Agarwal S.K., Loehr L., Mosley T., Alonso A. (2014). Atrial fibrillation and cognitive decline-the role of subclinical cerebral infarcts: The atherosclerosis risk in communities study. Stroke.

[10] Jefferson A.L., Liu D., Gupta D.K., Pechman K.R., Watchmaker J.M., Gordon E.A., Rane S., Bell S.P., Mendes L.A., Davis L.T. (2017). Lower cardiac index levels relate to lower cerebral blood flow in older adults. Neurology.

[11] Madhavan M., Hu T.Y., Gersh B.J., Roger V.L., Killian J., Weston S.A., Graff-Radford J., Asirvatham S.J., Chamberlain A.M. (2018). Efficacy of Warfarin Anticoagulation and Incident Dementia in a Community-Based Cohort of Atrial Fibrillation. Mayo Clin. Proc..

[12] Friberg L., Rosenqvist M. (2017). Less dementia with oral anticoagulation in atrial fibrillation. Eur. Heart J..

[13] Moffitt P., Park H., O’Connell J., Lane D.A., Quinn T.J. (2016). Thromboprophylaxis in atrial fibrillation and association with cognitive decline: Systematic review. Age Ageing.

[14] Díez-Villanueva P., Alfonso F. (2019). Atrial fibrillation in the elderly. J. Geriatr. Cardiol..

[15] Fuster V., Rydén L.E., Cannom D.S., Crijns H.J., Curtis A.B., Ellenbogen K.A., Halperin J.L., Le Heuzey J.-Y., Kay G.N., Lowe J.E. (2006). ACC/AHA/ESC 2006 Guidelines for the Management of Patients With Atrial Fibrillation-Executive Summary: A Report of the American College of Cardiology/American Heart Association Task Force on Practice Guidelines and the European Society of Cardiology Committee for Practice Guidelines (Writing Committee to Revise the 2001 Guidelines for the Management of Patients With Atrial Fibrillation). Eur. Heart J..

[16] Kirchhof P., Benussi S., Kotecha D., Ahlsson A., Atar D., Casadei B., Castellà M., Diener H.C., Heidbuchel H., Hendriks J. (2016). 2016 ESC Guidelines for the management of atrial fibrillation developed in collaboration with EACTS. Eur. Heart J..

[17] Freedman B., Potpara T.S., Lip G.Y.H. (2016). Stroke prevention in atrial fibrillation. Lancet.

[18] Gorelick P.B., Scuteri A., Black S.E., DeCarli C., Greenberg S.M., Iadecola C., Launer L.J., Laurent S., Lopez O.L., Nyenhuis D. (2011). Vascular contributions to cognitive impairment and dementia: A statement for healthcare professionals from the american heart association/american stroke association. Stroke.

[19] Subic A., Cermakova P., Religa D., Han S., Von Euler M., Kåreholt I., Johnell K., Fastbom J., Bognandi L., Winblad B. (2018). Treatment of Atrial Fibrillation in Patients with Dementia: A Cohort Study from the Swedish Dementia Registry. J. Alzheimer’s Dis..

[20] Wolf P.A., Abbott R.D., Kannel W.B. (1991). Atrial fibrillation as an independent risk factor for stroke: The Framingham Study. Stroke.

[21] Odutayo A., Wong C.X., Hsiao A.J., Hopewell S., Altman U.G., Emdin C. (2016). Atrial fibrillation and risks of cardiovascular disease, renal disease, and death: Systematic review and meta-analysis. BMJ.

[22] Rivard L., Khairy P., Talajic M., Tardif J.-C., Nattel S., Bherer L., Black S., Healey J.S., Lanthier S., Andrade J. (2019). Blinded Randomized Trial of Anticoagulation to Prevent Ischemic Stroke and Neurocognitive Impairment in Atrial Fibrillation (BRAIN-AF): Methods and Design. Can. J. Cardiol..

[23] Harada M., Van Wagoner D.R., Nattel S. (2015). Role of inflammation in atrial fibrillation pathophysiology and management. Circ. J..

[24] Barber M., Tait C., Scott J., Rumley A., Lowe G.D.O., Stott D.J. (2004). Dementia in subjects with atrial fibrillation: Hemostatic function and the role of anticoagulation. J. Thromb. Haemost..

[25] Marengoni A., Qiu C., Winblad B., Fratiglioni L. (2011). Atrial fibrillation, stroke and dementia in the very old: A population-based study. Neurobiol. Aging.

[26] Rastas S., Verkkoniemi A., Polvikoski T., Juva K., Niinisto L., Mattila K., Lansimies E., Pirttila T., Sulkava R. (2007). Atrial Fibrillation, Stroke, and Cognition: A Longitudinal Population-Based Study of People Aged 85 and Older. Stroke.

[27] Jacobs V., Woller S.C., Stevens S., May H., Bair T.L., Anderson J.L., Crandall B.G., Day J.D., Johanning K., Long Y. (2014). Time outside of therapeutic range in atrial fibrillation patients is associated with long-term risk of dementia. Heart Rhythm..

[28] Friberg L., Andersson T., Rosenqvist M. (2019). Less dementia and stroke in low-risk patients with atrial fibrillation taking oral anticoagulation. Eur. Heart J..

[29] Rash A., Downes T., Portner R., Yeo W.W., Morgan N., Channer K.S. (2007). A randomized controlled trial of warfarin versus aspirin for strokeprevention in octogenarians with atrial fibrillation (WASPO). Age Ageing.

[30] Subic A., Cermakova P., Norrving B., Winblad B., Von Euler M., Kramberger M.G., Eriksdotter M., Garcia-Ptacek S. (2017). Management of acute ischaemic stroke in patients with dementia. J. Intern. Med..

[31] Andreotti F., Rocca B., Husted S., Ajjan R.A., Berg J.T., Cattaneo M., Collet J.-P., De Caterina R., Fox K.A., Halvorsen S. (2015). Antithrombotic therapy in the elderly: Expert position paper of the European Society of Cardiology Working Group on Thrombosis. Eur. Heart J..

[32] Mant J., Hobbs F.D., Fletcher K., Roalfe A., Fitzmaurice D., Lip G.Y., Murray E., BAFTA investigators, Midland Research Practices Network (MidReC) (2007). Warfarin versus aspirin for stroke prevention in an elderly community population with atrial fibrillation (the BirminghamAtrial Fibrillation Treatment of the Aged Study, BAFTA): A randomised controlled trial. Lancet.

[33] Albers G.W., Dalen J.E., Laupacis A., Manning W.J., Petersen P., Singer D.E. (2001). Antithrombotic Therapy in Atrial Fibrillation. Chest.

[34] Tavassoli N., Perrin A., Berard E., Gillette S., Vellas B., Rolland Y., the REAL.FR Group (2013). Factors Associated with Undertreatment of Atrial Fibrillation in Geriatric Outpatients with Alzheimer Disease. Am. J. Cardiovasc. Drugs.

[35] Jankowska-Polanska B., Katarzyna L., Lidia A., Joanna J., Dudek K., Izabella U. (2016). Cognitive function and adher-ence to anticoagulation treatment in patients with atrial fibrillation. J. Geriatr. Cardiol..

